# Agatston scoring for assessment of coronary artery disease in patients undergoing transcatheter aortic valve implantation

**DOI:** 10.1007/s10554-025-03471-1

**Published:** 2025-08-01

**Authors:** Karim Mostafa, Jakob Christoph Voran, Markus Müller, Anka Pohlmeyer, Marie Noormalal, Mostafa Salem, Mohammed Saad, Patrick Langguth, Derk Frank, Carmen Wolf, Hatim Seoudy

**Affiliations:** 1 Department of Radiology and Neuroradiology, University Hospital Schleswig-Holstein, Campus Kiel, Kiel, Germany; 2 Department of Internal Medicine III, Cardiology and critical care, University Hospital Schleswig-Holstein, Campus Kiel, Kiel, Germany; 3 DZHK (German Centre for Cardiovascular Research), partner site Hamburg/Kiel/Lübeck, Kiel, Germany; 4 Department of Radiology and Neuroradiology, UKSH Kiel, Arnold-Heller-Straße 3, 24015 Kiel, Germany

## Abstract

Computed tomography (CT) angiography remains a cornerstone of pre-interventional assessment for transaortic valve implantation (TAVI). CT imaging prior to TAVI further allows for evaluation of the coronary arteries, with the simplest method being the calculation of the coronary Agatston score. Following the expansion of TAVI to lower risk patients, non-invasive coronary diagnostics in the framework of TAVI gain importance as they may allow patients to avoid unnecessary invasive coronary angiography (ICA) before the procedure. This study evaluates the diagnostic performance of coronary Agatston score values on pre-TAVI CT scans for the assessment of coronary artery disease. A total of 285 patients received a standard TAVI evaluation CT scan and routine ICA prior to the TAVI procedure. Agatston score values of the coronary tree and the proximal coronary segments (1, 5, 6, 11) were calculated separately by two radiologists. Reference for the AUC analysis was performance of PCI or stenting of the corresponding coronary segment following the recommendation of the Heart Team. ROC-analysis was performed for evaluation of the Agatston score at the level of the heart, the coronary arteries and the proximal coronary vessel segments (1, 5, 6 and 11) level to determine cutoff-values for optimal sensitivity and specificity. A total of 285 patients received a standard TAVI evaluation CT scan and routine ICA prior to the TAVI procedure. Agatston score values of the coronary tree and the proximal coronary segments (1, 5, 6, 11) were calculated separately by two radiologists. Reference for the AUC analysis was performance of PCI or stenting of the corresponding coronary segment following the recommendation of the Heart Team. ROC-analysis was performed for evaluation of the Agatston score at the level of the heart, the coronary arteries and the proximal coronary vessel segments (1, 5, 6 and 11) level to determine cutoff-values for optimal sensitivity and specificity. Coronary artery disease requiring intervention was present in 61 patients upon ICA (17.9%). In predicting the need of performance of a coronary intervention at a sensitivity of >89%, Agatston values of the full coronary tree >170 allow for a sensitivity of 90% (AUC 0.76, NPV 0.93). The sum Agatston score of the proximal segments >117 enables a sensitivity of 90% (AUC 0.74, NPV 0.93) and >47 a sensitivity of 98% (NPV 0.98). On the segmental level, Agatston values of >22 in the proximal RCA and >30 in the proximal LAD and CX allow for a sensitivity of 90% (AUC RCA 0.74, NPV 0.98; LAD 0.71, NPV 0.94; CX 0.75, NPV 0.99). Aiming for 98% sensitivity or higher, Agatston values of >2 for the RCA, >3 for the LAD and >27 for the CX apply (RCA NPV 1; LAD NPV 0.97; CX NPV 1). Coronary artery Agatston scoring on TAVI evaluation CT scans can serve as a predictor for the need of coronary interventions in the workup leading up to TAVI and it provides valuable information for identification of patients without significant coronary artery disease and may aid in allowing for the avoidance of invasive coronary artery diagnostics.

**Supplementary Information:**

The online version contains supplementary material available at 10.1007/s10554-025-03471-1.

## Introduction

Transcatheter aortic valve replacement (TAVI) is an established procedure where the aortic valve is replaced in a minimally invasive fashion. Over the course of the past decade, indications for TAVI have expanded to lower risk patients, emphasizing the importance of pre-interventional diagnostic workup for best-possible patient selection.

International guidelines on the management of valvular heart disease suggest that proximal coronary arterial stenosis be assessed and treated with percutaneous coronary intervention prior to TAVI (ESC, Class IIa, C) and ACC/AHA (Class 2a, C-LD)) [1, 2]. While interventional angiography remains the best possible means of vascular assessment, the disadvantages of it being an interventional procedure carrying risks and requiring arterial access, even if only performed with diagnostic intent, need to be considered [3]. Considering expansion of the indication for the TAVI procedure to lower risk patients, mandatory invasive coronary diagnostics may expose patients to non-benefiting risky diagnostic procedures while compromising safety and not upholding cost-effectiveness. Hence, the importance of best-possible non-invasive diagnostics as well as the screening-character of the pre-TAVI examinations, especially cardiac computed tomography, need to be reinforced.

Cardiac and thoracoabdominal computed tomography remain the gold standard imaging methods for planning of the TAVI procedure [1]. In the framework of cardiac CT imaging in patients undergoing TAVI, it has been debated as to whether assessment of the coronary arteries can be purposefully completed with CT and CTA [4–9]. However, standard TAVI-CT protocols are not dedicated for the detailed assessment of the coronary arteries by default and often do not allow for it. Furthermore, patients with severe symptomatic aortic stenosis in evaluation for TAVI cannot be adequately prepared for such coronary-specific CT with betablockers and nitroglycerin [9–11]. In recent years, different studies have covered the topic of coronary artery evaluation on TAVI-planning CT, however the results have not shown a clear trend. Most of these studies focused on diagnosis of significant coronary artery stenosis with often no regard as to whether treatment for the stenosis was performed [6, 8, 9, 12, 13].

The aim of this study was to investigate the value of coronary artery Agatston scoring on non-contrast cardiac CT scans as an independent marker for coronary artery disease in need of treatment in patients undergoing TAVI. Focus was put on Agatston analysis of the full coronary tree and a segment-by-segment analysis of the proximal coronary arteries.

## Materials and methods

This single centre retrospective study included all consecutive patients undergoing transfemoral TAVI between January 2018 and May 2022 at the University hospital Schleswig-Holstein in Kiel, Germany. Patients with insufficient visualization of the coronary arteries due to motion artefacts on CT imaging, in place coronary artery bypass, a coronary stent or with cardiac implantable electronic devices were excluded. A detailed consort flow diagram is included in the supplementary file. The study was approved by the ethics committee of the Christian-Albrechts-University in Kiel (Votum No. AZ D 506/22).

### CT imaging and imaging evaluation

CT imaging prior to the TAVI procedure was completed on one designated CT scanner (Definition Flash Siemens, Erlangen, Germany). Scanning parameters of the TAVI evaluation CT-imaging were standardised with the following parameters: tube voltage 100 kV, tube current time product 59 mAS, slice collimation 2.0 × 128 × 0.6 mm, pitch factor 1.2.

**Fig. 1:**
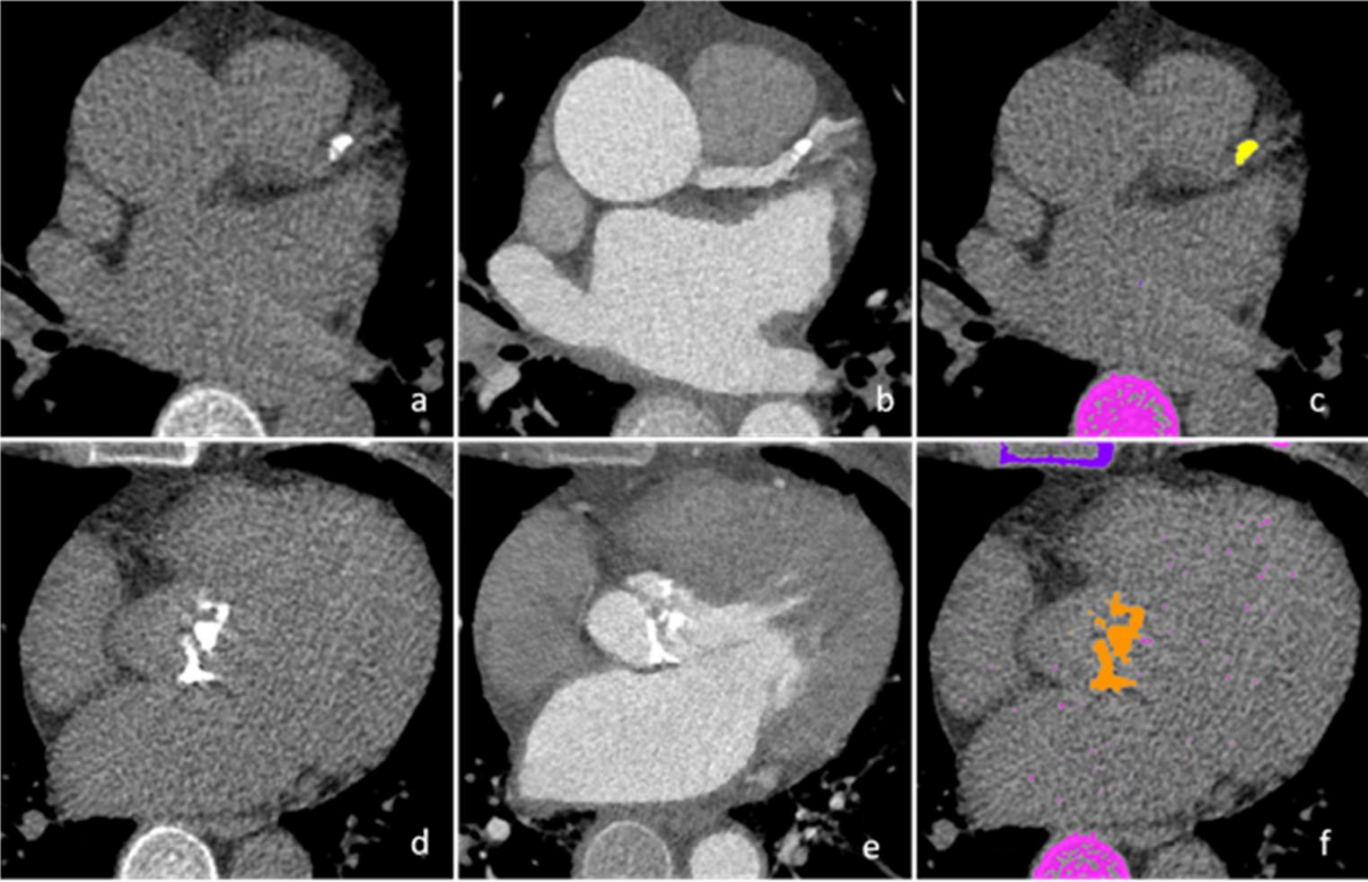
Graphic visualisation of measurements of Agatston-Score values on pre-TAVI CT imaging. On non-enhanced cardiac CT imaging (a, d), calcified lesions were identified and matched with the contrast-enhanced images (b, e). Afterwards, the calcified lesions were colour-coded and the Agatston-Score values were determined using imaging postprocessing software (SnygoVia, Siemens). In images a-c, evaluation of the LAD is visualised while in images e-f evaluation of the aortic valve is displayed

CT-imaging evaluation was completed in a two-step process. In the first step, non-enhanced imaging acquisition of the heart was completed for calculation of the Agaston-Score values of the full coronary tree, defined as overall Agatston score. In the second step, the Agatston score values were separately calculated for the proximal segments of the LAD, RCA and CX, whereby calcium scoring was performed for segments 1, 5, 6 and 11 [14]. Scoring was done manually by two radiologists with a designated assessment tool (C.W. and K.M., SyngoVia, Siemens, Erlangen, Germany; Fig. 1). Both radiologists were blinded to the results of the invasive coronary angiography.

### Invasive coronary angiography

All patients routinely underwent invasive coronary angiography (ICA) prior to the TAVI procedure. ICA was used to identify stenosis in need of potential treatment. The decision for treatment, whether interventional or conservative, was discussed in the multidisciplinary heart team considering the clinical and imaging findings. The heart team decision for stent placement was based on the location of the stenosis (proximal segments, left main trunk) and diameter (> 70% stenosis or > 50% for left main trunk).

### Statistics and data analysis

Data are presented as mean ± SD, median ± IQR or percentages as indicated. T-test or Mann-Whitney U test was applied for analysis of continuous variables while categorical data were analysed using the χ2 test or Fisher’s exact test. Statistical significance was considered given a two-sided p-value of less than 0.05. ROC-Analysis for Agatston values was conducted for the full coronary tree and the proximal coronary segments. Benchmark for the analysis was presence of a stenosis that received interventional treatment on all levels of assessment. Segmental analysis focussed on the proximal segments of the RCA (segment 1), the LAD (segment 5 and 6) and the CX (segment 11). Statistical analyses were done using R version 4.4.1.

## Results

### Patients

A total of 285 consecutive patients were included in the final analysis. Baseline demographic and clinical characteristics are summarized in Table 1. The cohort was divided into two groups – the “PCI group” included 61 patients in whom coronary angiography identified lesions in need of treatment and who subsequently underwent percutaneous coronary intervention. The “No PCI group” included 223 patients who showed no significant CAD in need of treatment upon invasive coronary angiography. Patients in the PCI group were significantly older (82.4 years vs. 80.4 years, *p* = 0.033; Table 1). Further, the proportion of male patients was higher in the PCI group (54% vs. 42%, *p* = 0.011; Table 1). Beyond age, no further significant baseline differences could be detected.

**Table 1:** Baseline Characteristics

**Characteristic**	**Overall**, N = 285 ^1^	**Intervention**		**p-value**^2^
		**No**, N = 224^1^	**Yes**, N = 61^1^	
Age	80.82 (6.28)	80.37 (6.45)	82.43 (5.37)	0.034
Sex, male	126 / 285 (44%)	93 / 224 (42%)	33 / 61 (54%)	0.011
BMI	27.60 (6.02)	27.69 (6.24)	27.42 (5.16)	0.8
Atrial Fibrillation/Flutter	110 / 285 (39%)	85 / 224 (38%)	25 / 61 (40%)	0.7
COPD	33 / 285 (12%)	27 / 224 (12%)	6 / 61 (11%)	0.6
Diabetes	80 / 285 (28%)	62 / 224 (28%)	18 / 61 (29%)	0.8
Dyslipidemia	174 / 285 (61%)	138 / 224 (62%)	36 / 61 (58%)	0.7
Hypertension	265 / 285 (93%)	208 / 224 (93%)	57 / 61 (94%)	>0.9
PAD	14 / 285 (4.9%)	10 / 224 (4.5%)	4 / 61 (6.5%)	0.5
Cerebrovascular disease	42 / 285 (15%)	31 / 224 (14%)	11 / 61 (18%)	0.4
History of smoking	49/285 (17,2%)	38 / 224 (17%)	11 / 61 (18%)	0.8
Family history for myocardial infarction^3^	8/285 (2,8%)	4 / 224 (1.8%)	4 / 61 (6.6%)	0.067
Baseline GFR (MDRD)	55.45 (20.34)	56.67 (20.41)	51.15 (19.65)	0.074
AVA	0.8 (0.2)	0.8 (0.2)	0.7 (0.2)	0.061
Reduced left ventricular ejection fraction (<55%)	73 / 248 (29%)	55 / 195 (28%)	18 / 53 (34%)	0.4

^1^Mean (SD); n / N (%)

^2^Wilcoxon rank sum test; Pearson's Chi-squared test; Fisher's exact test

^3^not systematically evaluated due to limited clinical relevance in an elderly TAVI cohort

### Capability of the Agatston score of the full coronary tree for prediction of relevant stenosis

Upon non-contrast enhanced cardiac CT, the PCI group showed higher total Agatston scores (3,286 vs. 5,092) and higher Agatston scores for the full coronary tree (303 vs. 1,313) with both results being significant (*p* < 0.001). The Agatston Score of the aortic valve alone was non-significantly higher in the PCI group (2,589 vs. 2,972, *p* = 0.056; Table 2).

**Table 2:** Characteristics of the coronary artery calcium score

**Characteristic**	**Overall**, N = 285^1^	**Intervention**		**p-value**^2^
		**No**, N = 224	**Yes**, N = 61^1^	
Agatston score total	3,523 (2,187, 5,445)	3,286 (2,068, 4,743)	5,092 (3,126, 7,019)	<0.001
Agatston score aortic valve	2,688 (1,675, 4,214)	2,589 (1,621, 4,070)	2,972 (1,848, 4,840)	0.056
Agatston score heart	430 (131, 1,142)	303 (85, 831)	1,313 (464, 2,626)	<0.001
Agatston score proximal segments	272 (83, 680)	193 (66, 498)	675 (312, 1,625)	<0.001
LMT Agatston score	18 (0, 96)	9 (0, 72)	80 (11, 217)	<0.001
LAD Agatston score	90 (20, 242)	66 (10, 199)	260 (77, 438)	<0.001
LCX Agatston score	32 (0, 132)	24 (0, 103)	84 (9, 277)	<0.001
RCA Agatston score	36 (1, 199)	25 (0, 110)	155 (33, 449)	<0.001

^1^Median (IQR)

^2^Wilcoxon rank sum test

**Fig. 2:**
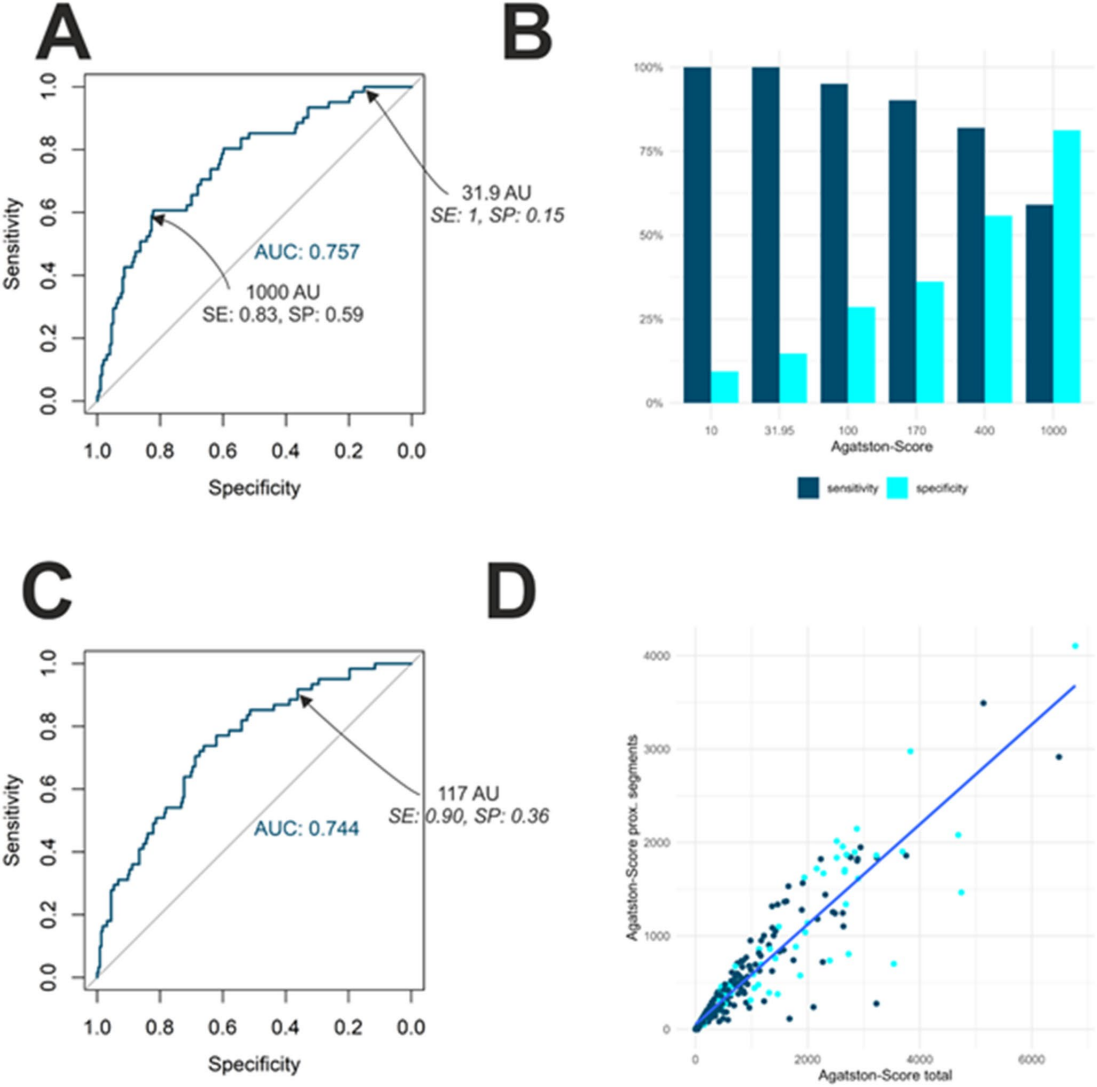
ROC-Analysis, Sensitivity and Specificity values for prediction of PCI prior to TAVI. **A**: Receiver operating characteristic (ROC) for Agatston values of the full coronary tree, **B**: Sensitivity and specificity for selected thresholds of interest of the full heart, **C**: Receiver operating characteristic for the sum Agatston Score of the proximal coronary arteries. **D**: Correlation of full heart Agatston score and sum Agatston score of the proximal segments (dark blue: “No PCI group”, light blue: “PCI group” AUC: area under the curve, SE: Sensitivity, SP: Specificity,

**Fig. 3:**
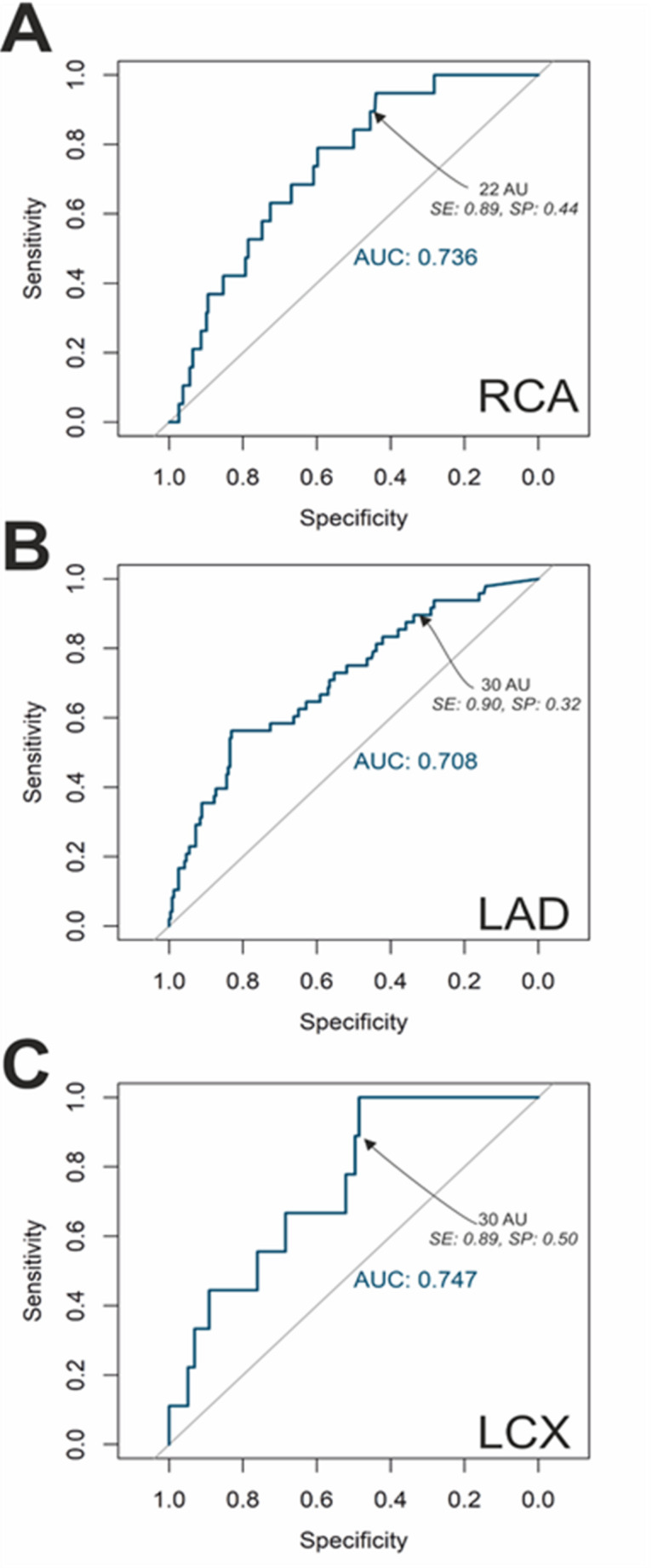
ROC-Analysis for determination of threshold Agatston values of the RCA, LAD and LCX. Abbreviations: AUC: area under the curve, LAD: Left anterior descending coronary artery, LCX: Circumflex coronary artery, RCA: Right coronary artery, SE: Sensitivity, SP: Specificity,

Considering the full coronary tree, we found an area under the curve of 0.76 (95% CI 0.69–0.82) for prediction of a stenosis in need of treatment in the ROC. Following the Youdens Index, the threshold for overall coronary artery score values with highest sensitivity and specificity was at 981 with a sensitivity of 80% and a specificity of 60%. Considering an overall Agatston value of 170, sensitivity will be at 90% with specificity at 36%. This result is presented in Figs. 2, 3; Table 3. Choosing this sensitivity would avoid ICA in 31% of patients in our cohort. A sensitivity of over 98% (Table 4) leads to a lower proportion of patients in whom an ICA could be potentially avoided.

**Table 3:** Different Agatston Score values and locations for prediction of a coronary stenosis in need of treatment with a target sensitivity of 89% and higher

**Agatston Score Location**	**Agatston Threshold Value**	**Sensitivity**	**Specificity**	**NPV**	**PPV**	**PNIA**
Overall Coronary Tree	170	90%	36%	93%	28%	31%
Proximal Segments (1, 5, 6, 11)	117	90%	36%	93%	28%	31%
RCA (Segment 1)	22	89%	44%	98%	10%	42%
LAD (Segments 5 and 6)	30	90%	32%	94%	21%	29%
CX (Segment 11)	30	89%	50%	99%	5%	48%

CX: Circumflex coronary artery, LAD: Left anterior descending coronary artery, NPV: negative predictive value; PNIA: Percentage of patients with a **P**otential **N**on-**I**nvasive **A**ssessment prior to TAVI (of the respective segment), PPV: positive predictive value, RCA: Right coronary artery

**Table 4:** Different Agatston Score values and locations for prediction of a coronary stenosis in need of treatment with a target sensitivity of 98% and higher

**Agatston Score Location**	**Agatston Threshold Value**	**Sensitivity**	**Specificity**	**NPV**	**PPV**	**PNIA**
Overall Coronary Tree	50	98%	18%	98%	25%	14
Proximal Segments (1, 5, 6, 11)	47	98%	20%	98%	25%	16
RCA (Segment 1)	2	99%	28%	100%	9%	26
LAD (Segment 5 and 6)	3	98%	14%	97%	19%	12
CX (Segment 11)	27	99%	49%	100%	6%	47

CX: Circumflex coronary artery, LAD: Left anterior descending coronary artery, NPV: negative predictive value; PNIA: Percentage of patients with a **P**otential **N**on-**I**nvasive **A**ssessment prior to TAVI (of the respective segment), PPV: positive predictive value, RCA: Right coronary artery

### Agatston score analysis for sum scores of the proximal segments of LAD, CX and RCA

The heart team’s recommendation for stents was made for proximal stenoses, as these were considered to be particularly prognostically relevant. We therefore performed the analysis for the Agatston scores of the proximal segments in order to examine whether this would affect the predictive power of the results.

The sum of the Agatston scores of the proximal coronary segments was significantly lower in the “No PCI group” compared to the “PCI group” (165 vs. 193, *p* < 0.001). ROC analysis has shown an AUC of 0.74 for prediction of a stenosis in need of treatment. Considering the threshold of 117, sensitivity will be at 90% with a specificity of 36% (Table 3). The negative predictive value (NPV) is at 0.93. When using a sensitivity threshold of 98%, the Agatston score threshold is placed at 47 with an NPV of 0.98) (Table 4). In both cases the predictive quality was comparable to the Agatston score of the full coronary tree.

### Segmental Agatston scoring for proximal segments of LAD, RCA and CX

Patients in the “No PCI” group showed significantly lower Agatston score values for proximal segments of the LAD (segment 5 and 6, 66 vs. 260, *p* < 0.001), RCA (segment 1, 25 vs. 255, *p* < 0.001) and CX (segment 11, 24 vs. 84, *p* < 0.001), respectively. ROC analysis for prediction of a relevant stenosis of the RCA (segment 1) showed an AUC of 0.74. At the score value of 22, a sensitivity of 95% could be achieved at a specificity of 44% (NPV 0.98). For the LAD (segment 5 and 6), we found an AUC of 0.71, whereby a sensitivity of 90% could be achieved when using the score value 30 as threshold, however specificity remained low at 32% (NPV 0.94). Finally, for the CX (segment 11), we calculated an AUC of 0.75. Here we found a sensitivity of 89% at a specificity of 50% at the threshold value of 30 (NPV 0.99; Table 3).

When considering a sensitivity threshold of 98% or higher, the threshold for the RCA (segment 1) is at 2 (NPV 1), for the LAD (segment 5 and 6) at 3 (NPV 0.97) and for the CX (segment 11) at 27 (NPV 1; Table 4).

### Performance of the Agatston score of the entire coronary tree for the prediction of stenoses

#### > 70% without consideration of need for PCI

In 26 patients we found stenoses > 70% at any coronary artery in the “No PCI group”. Reasons for not intervening these stenoses were small insignificant vessels or a chronic total occlusion without a benefit of intervention. In one patient PCI was avoided as the bleeding risk associated with the dual antiplatelet therapy was considered unfavourable in view of an imminent urgent operation. We reanalysed the data set with > 70 stenoses as a benchmark to obtain comparable data and minimise bias. This resulted in an area under the curve of 0.72 (95% CI 0.65–0.78) (Supplementary Fig. 2). The Agatston score value to archive a sensitivity of 98% was slightly lower than for prediction stenoses that underwent PCI, hence the number of patient where ICA could be avoided was lower (Supplementary Table 2).

## Discussion

The main goal of this study was the assessment of potential role of the Agatston score of the full coronary tree and proximal coronary segments as independent marker for coronary artery disease in need of interventional treatment in patients undergoing TAVI. Furthermore, for the first time this study provides data on proximal coronary artery segment Agatston score values in patients undergoing workup for TAVI.

The main findings of our study are: (1) Overall, patients in need of percutaneous coronary intervention prior to TAVI showed significantly higher Agatston Score values for the full coronary tree and the proximal coronary artery segments compared to patients who did not need a coronary intervention. (2) Considering a threshold Agatston score value of 22 in the proximal RCA and 30 in the proximal LAD and CX as well as a sum score of the proximal segments of > 117 allowed for a sensitivity ≥ 90% for prediction of a stenosis in need of interventional treatment. (3) Agatston values of the full coronary tree of > 170 allowed for a sensitivity of 90% for prediction of need of a percutaneous coronary intervention.

### Prediction of coronary stenosis in need of treatment

In our study, we showed that full coronary tree as well as individual and combined proximal coronary artery Agatston score values allow for screening of a stenosis in need of treatment at high sensitivity levels of ≥ 90%. Overall, the performance of the ROC analysis results can be classified as acceptable [15]. The suggested cutoff values are 170 for the full coronary tree, 117 for the sum score of the proximal segments, 22 for the RCA (segment 1) and 30 for the LAD (segment 5 and 6) and CX (segment 11), respectively. The selected sensitivity is worthy of discussion and could be incorporated into a shared decision-making process with the patient concerned. A higher sensitivity would increase the certainty of not missing a stenosis but would lead to higher rates of invasive coronary angiography. In our opinion, a cut-off corresponding to a sensitivity of 90% is reasonable, as the evidence for stent implantation in patients, particularly without chest pain, is generally limited [16, 17].

In 2022, Malebranche et al. have extensively addressed this topic in their work, reporting an AUC of 0.75 for Agatston scoring of the coronary tree for prediction of a stenosis > 50%, which is in good agreement to our results [9]. However, this study used stenosis > 50% as benchmark, while we focused on stenoses that went on to receive interventional treatment, rendering our data more real-world applicable. While their study used Agatston values mainly as add-on to CTA to enhance diagnostic performance, our study further encompasses a segmental analysis of the coronary arteries with the Agatston Score which has not yet been done before. Further, their study suggests a cutoff of > 100 for the full coronary tree, which is comparable to our suggested cutoff of 170 [9]. Gohmann et al. 2024 also suggested an Agatston cutoff value of 110 for exclusion of CAD in their study of 460 patients prior to TAVI, which is in good agreement to our findings [13].

### Role of the Agatston score on non-contrast CT vs. coronary CTA on TAVI evaluation CT

In the past decade a multitude of research items has focused on assessment of coronary CTA in the framework of TAVI evaluation CT imaging [3, 7–10, 13]. In a meta-analysis by Gatti et al. in 2022, the authors have found coronary CTA in this framework to be an excellent diagnostic tool for exclusion of coronary artery disease [18]. While the potential of coronary CTA in this specific framework is promising, relevant diagnostic limitations arise during image acquisition due to often extensive coronary calcifications and the aforementioned lack of heart-rate control and beta-blocker admission. This problem has to be considered in this framework and it is reflected in the published data, since in both recent and past research items non-evaluable coronary CT studies are mentioned in up to 20–84% of cases, representing a major diagnostic disadvantage [7, 9, 12, 13]. These issues are also discussed in a meta-analysis of 7458 patients in 2024 [19]. The Agatston score, which is not affected by the acquisition limitations of CTA, can be a useful tool in this setting. Especially in the framework of artifact-impaired or non-diagnostic studies, our suggested cutoff values of 170 for the full coronary tree and 117 for the proximal segments allow for a more detailed assessment of coronary artery disease. Malebranche et al. 2022 have incorporated the Agatston score into CTA analysis in their study of 100 patients showing an increase in specificity of 18–39% depending on the applied cutoff value of 400 or 100 for the overall coronary artery Agatston score [9]. The suggested cutoff values for the proximal coronary segments of our study of > 22 for the RCA and > 30 for the LAD and CX will allow for more comprehensive diagnostics in the per segment analysis and can counter the opposing limits in a CTA only approach.

### Considerations of non-contrast imaging prior to TAVI

One of the main advantages of prediction of coronary artery disease with Agatston scoring is the avoidance of application of contrast media. Especially in the framework of Agatston scoring of the aortic valve, scoring of the coronary arteries can be done simultaneously. A possible application for this workflow is represented in patients with impaired renal function. Here, after non-contrast enhanced Agatston scoring further evaluation of the TAVI procedure can be performed with non-contrast MRI as described in multiple research items [20–23].

### Limitations

This is a retrospective study with its associated limitations. This cohort only included patients who underwent TAVI evaluation with subsequent placement of a TAVI. With our patient collective being clearly defined, a large number of patients had to be excluded due to presence of metal artefacts and existing bypasses, which overall lowers the generalizability of our findings and has to be considered when interpreting our results. Furthermore, while our study shows promising results for coronary artery assessment with the Agatston score, both these findings and the potential benefit of segmental analysis combined with CTA should be further investigated in larger cohorts. It is important to mention that image acquisition was done with a tube voltage of 100 kVp, which may lead to an underestimation of Agatston score values. Lastly, Agatston scoring does not allow for the evaluation of non-calcified coronary artery plaques, which represents a limitation of the modality [24].

## Conclusion

Agatston scoring on pre-TAVI CT of the full coronary tree and proximal coronary segments shows promise in predicting the need for percutaneous coronary intervention and in identifying patients who may safely forgo invasive coronary angiography. Although image artifacts limited assessability in a significant portion of cases, the consistent findings in evaluable scans support the potential clinical utility of this approach).

## Supplementary Information

Below is the link to the electronic supplementary material.

Supplementary file1 (DOCX 154 KB)

## Data Availability

No datasets were generated or analysed during the current study.

## Abbreviations

CT: Computed tomography
CTA: Computed tomography angiography
ICA: Invasive coronary angiography
TAVI: Transcatheter aortic valve implantation
ROC: Receiver operating characteristics
AUC: Area under the curve
LAD: Left anterior descending coronary artery
CX: Circumflex coronary artery
RCA: Right coronary artery
