# Supplementary material for: Agatston scoring for assessment of coronary artery disease in patients undergoing transcatheter aortic valve implantation
Source: Int J Cardiovasc Imaging. 2025 Aug 1;41(9):1729–38. doi: 10.1007/s10554-025-03471-1 (PMC12405386; doi:10.1007/s10554-025-03471-1)
Supplement: Supplementary file 1 — Supplementary file1 (DOCX 154 KB) [file 10554_2025_3471_MOESM1_ESM.docx]

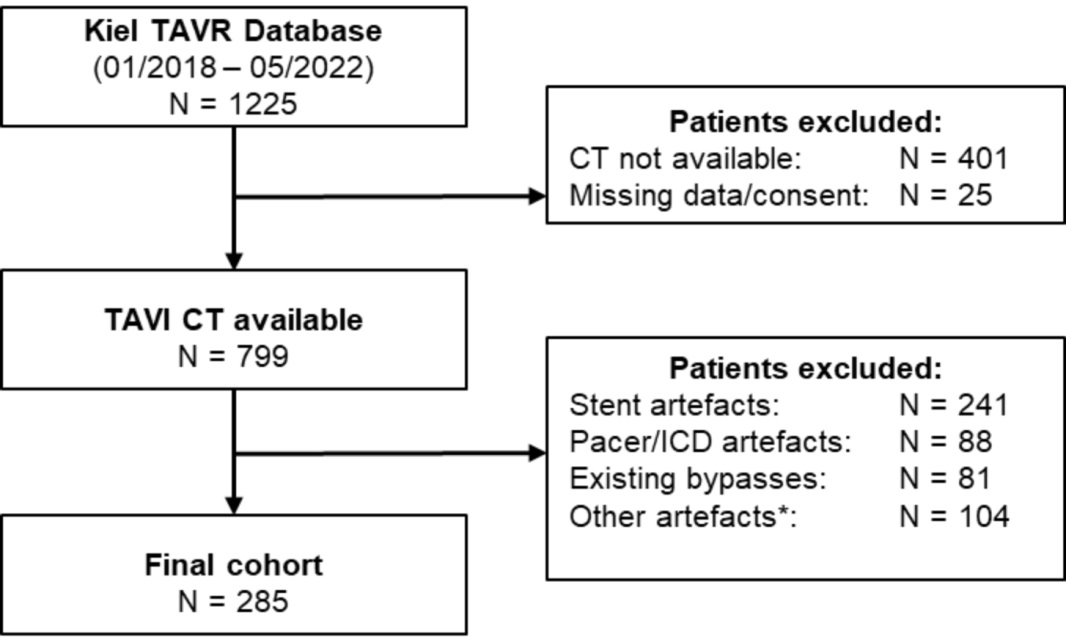


**Supplementary Fig. 1.** Consort flow diagram

*Other artefacts include patients with cardiac implantable devices (e.g. left atrial appendage occlusion device, prior aortic valve replacement, other prior cardiac surgery), movement artefacts and pulsation artefacts

**
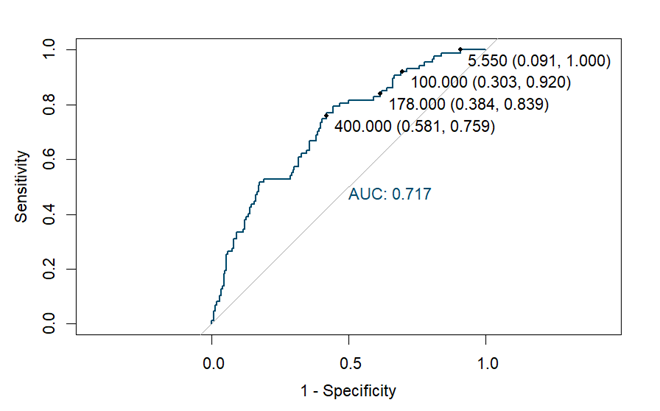
**

**Supplementary Fig. 2. ROC-Analysis for prediction of a 70% coronary artery prior to TAVI**

**Supplementary Table 1.** Angiographic characteristics of patients receiving PCI.

| **Invasive coronary angiography findings** | | **N = 61** |
| --- | --- | --- |
| Left anterior descending artery > 70% | | 49/61 (80) |
| Circumflex artery > 70% | | 26/61 (43) |
| Right coronary artery > 70% | | 24/61 (39) |
| Left main stem coronary artery > 50% | | 16/61 (26) |
| Chronic total occlusion | | 2/61 (3) |
| Number of vessels intended for PCI | 1 | 30/61 (49) |
|  | 2 | 19/61 (31) |
|  | 3 | 11/61 (18) |
|  | 4 | 1/62 (2) |
| Number of Stents/patient | 1 | 17/61 (28) |
|  | 2 | 23/61 (38) |
|  | ≥ 3 | 21/61 (34) |
| Number of treated lesions | 1 | 30/61 (49) |
|  | 2 | 18/61 (30) |
|  | ≥ 3 | 13/61 (21) |

N (%)

**Supplementary Table 2:** Different Agatston Score values and locations for prediction of a coronary stenosis > 70% with a target sensitivity of 98% and higher.

|  | **Agatston Threshold Value** | **Sensitivity** | **Specificity** | **NPV** | **PPV** | **PNIA** |
| --- | --- | --- | --- | --- | --- | --- |
| Total coronary tree | >=44 | 0.98 | 0.19 | 0.95 | 0.35 | 14 |

NPV: negative predictive value; PNIA: Percentage of patients with a **P**otential **N**on-**I**nvasive **A**ssessment prior to TAVI (of the respective segment), PPV: positive predictive value
